# Genomic and transcriptomic analyses of *Citrus sinensis* varieties provide insights into Valencia orange fruit mastication trait formation

**DOI:** 10.1038/s41438-021-00653-5

**Published:** 2021-10-01

**Authors:** Guizhi Feng, Xiu Ai, Hualin Yi, Wenwu Guo, Juxun Wu

**Affiliations:** Key Laboratory of Horticultural Plant Biology, Ministry of Education, Huazhong Agricultural University, Wuhan, PR China

**Keywords:** Natural variation in plants, Plant development

## Abstract

Valencia orange (*Citrus sinensis* Osbeck) (VO) is a type of late-ripening sweet orange whose ripening occurs 4 to 5 months later than that of the mid-ripening common sweet orange (CO). Notably, the mastication trait of VO fruit is inferior to that of CO fruit. To date, how inferior pulp mastication trait forms in VO has not been determined. In this study, 13 VO varieties and 12 CO varieties were subjected to whole-genome resequencing. A total of 2.98 million SNPs were identified from 25 varieties, and a SNP molecular marker was developed to distinguish VO and CO. Moreover, 144 and 141 genes identified by selective sweep analysis were selected during VO and CO evolution, respectively. Based on gene functional enrichment analysis, most of the selected VO genes were related to the stress response and lignin biosynthesis. Simultaneously, we comparatively analyzed the transcriptome profiles of peel and pulp tissues among three VO varieties and three CO varieties, and the results demonstrated differences in lignin biosynthesis between VO and CO fruits. Furthermore, coexpression network analysis was performed to identify hub genes of lignin-related and variety-specific networks, which included *CsERF74*, *CsNAC25*, *CsHSFB3*, *CsSPL4/13*, etc. Overall, this study provides important insights into the mastication trait formation of Valencia orange fruit.

## Introduction

Sweet orange (*Citrus sinensis* Osbeck) is widely cultivated worldwide and has various varieties. Valencia oranges (VOs) constitute an important group of natural late-ripening sweet orange varieties, whose maturation stage is delayed by 4–5 months compared to that of other common sweet orange (CO) varieties^1^. Fruit mastication trait is an important index for evaluating citrus fruit quality and directly influences the quality of fruit commodities. It is known that the mastication trait of VO fruit is inferior, which greatly affects its quality. However, the regulatory mechanism underlying the formation of VO fruit mastication trait is unclear.

The mastication trait of citrus fruit is related to dietary fiber properties of the fruit pulp, which are related to cell wall metabolism. Pectin, cellulose, and lignin are important components of the cell wall^2,3^. Many enzymes play roles in cell wall metabolism; these enzymes include polygalacturonase (PG), pectin methyl esterase (PME) and lignin biosynthesis-related genes (PALs, C4Hs, 4CLs, CCoAOMTs, CADs, etc.)^4^. Many studies have shown that the accumulation of lignin in fruit can affect fruit texture and quality. Lignin is deposited in large amounts in the peel and flesh of pear, resulting in the formation of rough-textured flesh^5^. Under 0 °C storage conditions, the accumulation of lignin in peach fruit diminishes their taste^6^. In citrus, the accumulation of lignin can reduce the water in juice cells and further lead to fruit granulation, which causes inferior fruit mastication trait^7^. In addition, lignin plays essential roles in supporting plants, water transport, and resistance to external stress factors (e.g., cold stress)^8–11^.

Lignin is biosynthesized through the phenylpropane metabolic pathway, which includes three main processes: biosynthesis, transport, and polymerization of monoxylin^12^. Several key metabolic genes have been identified in the phenylpropane metabolic pathway, such as *PAL*, *C4H*, *4CL*, *CCoAOMT*, *CCR*, *F5H,* and *CAD*^4^. Plant lignin consists mainly of G, S, and H units, among which gymnosperm lignin is composed mainly of G and H units, and dicotyledonous angiosperm lignin is composed mainly of G and S units^13^. In general, cell walls undergo lignification during plant stress^14^. Several studies have shown that salt stress^15^, boron deficiency stress^16^, and cold stress^17^ enhance lignin production.

Selective sweep analysis is used to identify footprints of species that have experienced strong positive natural or artificial selection during evolution^18,19^. A population differentiation (*F*_*st*_) and nucleotide diversity level (θ_π_) based cross approach has been shown to be very effective for detecting selective elimination regions, especially when functional regions closely related to the living environment are mined, and the two approaches can jointly identify strong selection signals, which facilitates the screening of target genes^20^. This approach has been applied to numerous organisms (including both plants and animals) to study the footprints of positive selection in genomes. For instance, *F*_*st*_ and θ_π_ have been used to detect selective signals during grapevine domestication, and nine selective sweep genomic regions were identified^21^. Similarly, selective sweep analysis revealed the genomic regions of Berkshire, a native European pig, related to disease resistance, pork production, fertility, tameness and body length^22^. Moreover, several studies have shown that the identification of genomic region selection signatures is an effective approach associated with horticultural traits of fruit species (e.g., grape^21^, citrus^23^, pear^24^, and peach^25,26^).

In this study, 13 VO varieties and 12 CO varieties were sampled for genome resequencing, and three VO varieties and three CO varieties were subjected to RNA-seq analysis. Genomic and transcriptomic analyses were integrated to provide insights into VO fruit mastication trait formation.

## Results

### Detection of genetic variations in VOs and COs

**Fig. 1:**
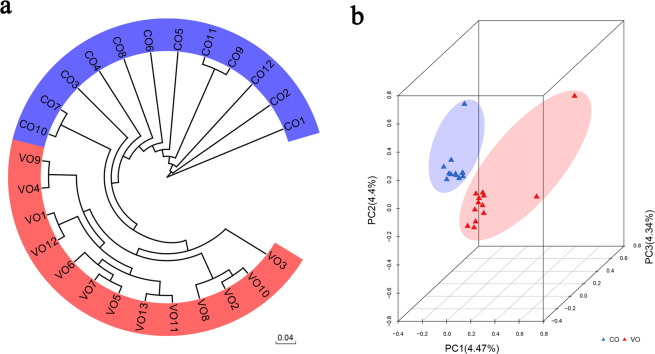
Analysis of the population genetic diversity of 25 sweet orange varieties. **a** Neighbor-joining tree of all the varieties, constructed using SNP resequencing data. Scale bar: 0.04. **b** 3D scatter plot of 25 varieties according to individual SNP differences via principal component analysis (PCA). The two shaded areas show the different subgroups: red corresponds to VOs, and blue corresponds to COs

A total of 25 sweet orange varieties (13 VOs and 12 COs) were selected for genome resequencing. The 25 varieties are different among each other in terms of fruit size, fruit color, number of seeds, maturity, and place of origin, among other characteristics (Supplementary Table S1). Genome resequencing was performed with an average depth of 14 ± 3× and an average genome coverage of 95 ± 3% (Supplementary Table S1). A total of 2.98 million SNPs were identified in the 25 varieties. Most SNPs (52.4%) were distributed in intergenic regions (Supplementary Fig. S1, Supplementary Table S2). A neighbor-joining tree was constructed to visualize the pairwise genetic distances of VOs and COs, which revealed that VOs and COs could obviously be divided into two groups (Fig. 1a). Subsequently, principal component analysis (PCA) showed that most of the samples fell in the two clouds, but three samples (CO10, VO1, and VO4) were distant from the others (Fig. 1b).

### Genome-wide molecular footprints of selections

**Fig. 2:**
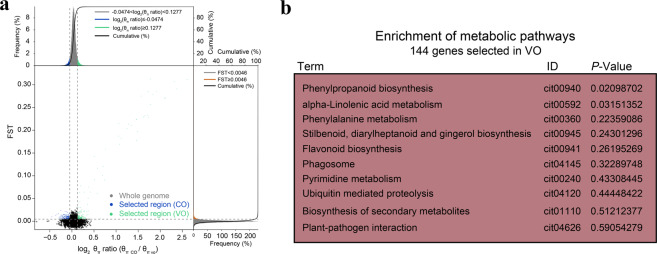
Selective sweep genomic regions and functional analysis of selected genes. **a** Distribution of θ_π_ ratios (log_2_θ_π_, CO/log_2_θ_π_, VO) and *F*_*st*_ values. The horizontal and vertical dashed lines mark the regions in the top 5% of *F*_*st*_ values and θ_π_ ratios, respectively. The blue points represent selected regions in the CO genome, and the green points represent selected regions in the VO genome. **b** Top 10 enriched KEGG pathways of selected genes from VO

To detect the genomic molecular footprints left by natural and artificial selection, 25 cultivated accessions (13 VOs and 12 COs) representing the major varieties of VOs and COs were selected for population analysis. As shown in Supplementary Fig. S2a, the Tajima’s D values of VO and CO were both far from zero, which indicated that both the VO and the CO groups were subjected to positive selection (including balancing selection and directional selection). According to an analysis of linkage disequilibrium (LD), the LD decay distance of VO was very close to that of CO (Supplementary Fig. S2b). Selective sweep analysis was then used to identify the selected regions of the genome^27^. An approach based on *F*_*st*_ and θ_π_ values was used to identify the selective sweep regions in the VO and CO genomes. As shown in Fig. 2a, 29 and 105 selective sweep regions with significant signals were identified within CO and VO, respectively.

Among the COs, 141 genes were contained in the 29 selected regions. Of these genes, 14 disease resistance proteins, 3 myrcene synthases, and 8 leucine-rich repeat- containing proteins were identified (Supplementary Table S4). The set of these genes was related mainly to positive regulation, stimulus response, and defense response (Supplementary Fig. S3a; Supplementary Table S5). According to the results of the KEGG enrichment analysis, there were no enriched pathways (Supplementary Table S6).

In the VOs, 144 genes were identified from 105 selected regions. These regions harbored 15 disease resistance proteins, 2 probable WRKY transcription factors, and 10 leucine-rich repeat-containing proteins. Notably, 6 phenylpropanoid biosynthesis-related genes, such as caffeic acid 3-O-methyltransferase (e.g., orange1.1t05218, Cs5g24980, and Cs5g19020) and caffeoyl-CoA O-methyltransferase (Cs1g22450), were identified in VO (Supplementary Table S4). The set of these 144 genes was related mainly to lignin biosynthesis, defense response, stimulus response, and developmental regulation (Supplementary Fig. S3b). Furthermore, several enriched pathways were identified in VOs, including the ‘phenylpropanoid biosynthesis’ and ‘alpha-linolenic acid metabolism’ pathways (Fig. 2b; Supplementary Table S6). Taken together, these results indicated that lignin metabolism-related genes may contribute to the physiological differences between VOs and COs.

### Transcriptome profiles revealed differential regulation in VO and CO fruits

To further explore the differences in the molecular regulation between VO and CO, we performed a comparative fruit transcriptome analysis of three VO varieties (Cutter Valencia orange, Delta Valencia orange and Rohde Red Valencia orange) and three CO varieties (Jincheng orange, Taoye orange, and Xianfeng orange), which are typical representative varieties of VO and CO, respectively. The fruits of these six sweet orange varieties were collected, which includes two tissue types (peel and pulp), at 220 days after flowering (DAF). Data for a total of 36 transcriptomes were obtained from 12 samples (three biological replicates per sample) (Supplementary Table S7). A correlation dendrogram and PCA illustrated good global relationships among different CO/VO groups and peel/pulp tissues (Supplementary Fig. S4a, b).

**Fig. 3:**
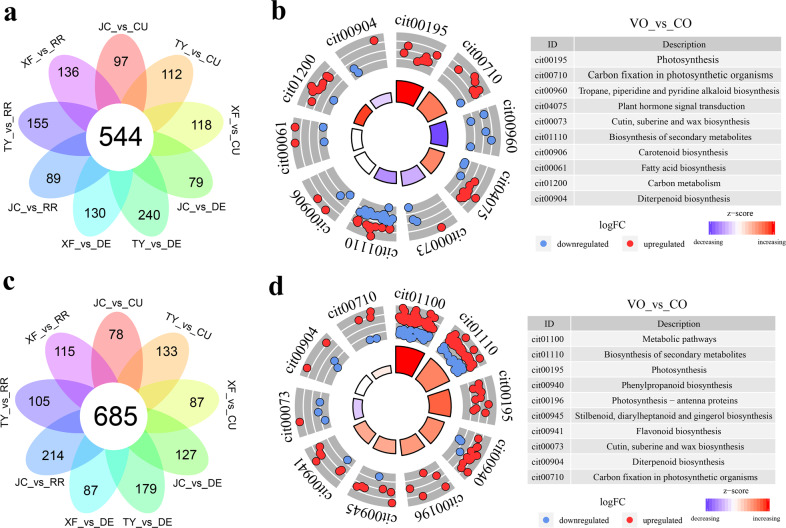
Identification and functional enrichment of DEGs between VO and CO. Flower plot diagrams of DEGs in the peel (**a**) and pulp (**c**) between VO and CO. In all subsequent figures, TY means Taoye orange, JC means Jincheng orange, XF means Xianfeng orange, CU means Cutter Valencia orange, DE means Delta Valencia orange, and RR means Rohde Red Valencia orange. KEGG pathway enrichment analysis of DEGs in the peel (**b**) and pulp (**d**). logFC means log_2_ (ReadCount_ VO/ReadCount _CO)

As shown in Fig. 3a, c, 544 and 685 differentially expressed genes (DEGs) were identified in peel and pulp tissue, respectively, by pairwise comparison of CO and VO samples. Then, several DEGs were randomly selected to verify their expression via qRT-PCR, the results of which were in accordance with the RNA-seq results (Supplementary Fig. S4c). Subsequently, GO enrichment analysis revealed that ‘response to stimulus’ and ‘lipid metabolic process’ were enriched in the peel, whereas ‘response to stimulus’ and ‘phenylpropanoid biosynthetic process’ were enriched in the pulp (Supplementary Fig. S5a and b). Moreover, KEGG enrichment analysis revealed that ‘plant hormone signal transduction’, ‘carotenoid biosynthesis’ and ‘fatty acid biosynthesis’ were enriched in the peel, whereas ‘phenylpropanoid biosynthesis’ and ‘flavonoid biosynthesis’ were enriched in the pulp. Importantly, most genes in the ‘phenylpropanoid biosynthesis’ pathway were upregulated in VOs (Fig. 3b, d). Taken together, these results indicated that these genes may contribute to the accumulation of lignin in the pulp of VOs, which may result in inferior fruit mastication trait of VOs.

### Coexpression network analysis reveals regulatory programs involved in lignin metabolism

Coexpression networks were constructed using the WGCNA package^28^ based on the RNA-seq data. After excluding genes with low expression (FPKM < 0.3), a total of 16,328 genes were categorized into 18 modules (Supplementary Fig. S6a, b).

**Fig. 4:**
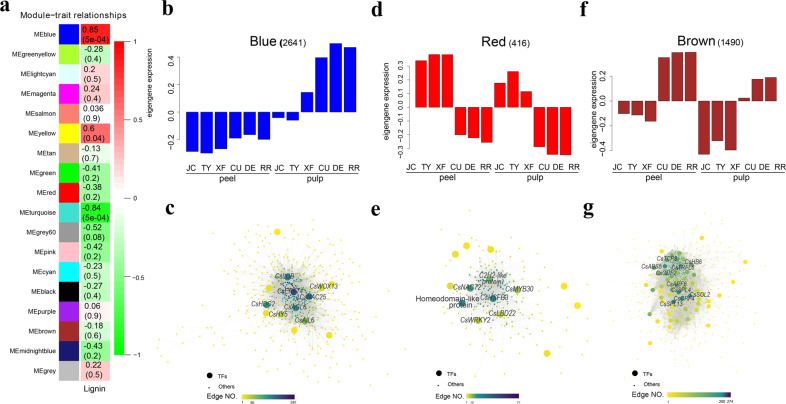
Functions and networks of coexpression module genes. **a** Module-trait relationships. The rows indicate the different modules, and the columns indicate lignin. The red and green colors indicate positive and negative correlations, respectively. The correlation coefficient and *P*-value are displayed in each cell. **b**, **d**, **f** Eigengene expression profiles in the blue, red, and brown modules. **c**, **e**, **g** Construction of the correlation network of the blue, red and brown modules. The large circles represent transcription factors. The color is determined by the edge number of the gene

We evaluated the correlations between gene modules and the lignin content using WGCNA. Lignin was highly correlated with the blue module (R^2^ = 0.85, *P* = 5e-4) (Fig. 4a). The genes of the blue module were significantly upregulated in the pulp of VOs and were mainly enriched in ‘lignin biosynthetic process’ and ‘response to cold’ processes (Fig. 4b; Supplementary Fig. S7a). In addition, two CO- and VO-specific modules (red, 416 genes; brown, 1,490 genes) were further analyzed (Fig. 4d, f). The two sets of genes from the red and brown modules were subjected to GO term enrichment analysis (Supplementary Table S5). The genes of the red module were significantly enriched in the biological processes ‘response to abscisic acid’ and ‘response to oxidative stress’ (Supplementary Fig. S7b). In the brown module, genes were significantly enriched in the ‘response to cold’, ‘response to wounding’ and ‘lignin biosynthetic process’ biological processes (Supplementary Fig. S7c).

To identify the key regulatory genes in the blue, red, and brown modules, we constructed gene networks via WGCNA and Cytoscape. Ultimately, 14 of 581 genes, 14 of 205 genes, and 61 of 922 genes encoded transcription factors (TFs) in the blue, red, and brown module networks, respectively. For instance, *CsERF74* (Cs1g16690) was the hub gene in the blue module network and had the highest number of edges (178 edges) (Fig. 4c). In other module networks, the highly connected hub TFs included *CsNAC25* (Cs2g06460), *CsHSFB3* (orange1.1t02319), *CsSPL4/13* (Cs2g23550/Cs7g10990), etc. (Fig. 4c, e and g).

### Identification of key genes involved in cell wall modification in citrus fruit

**Fig. 5:**
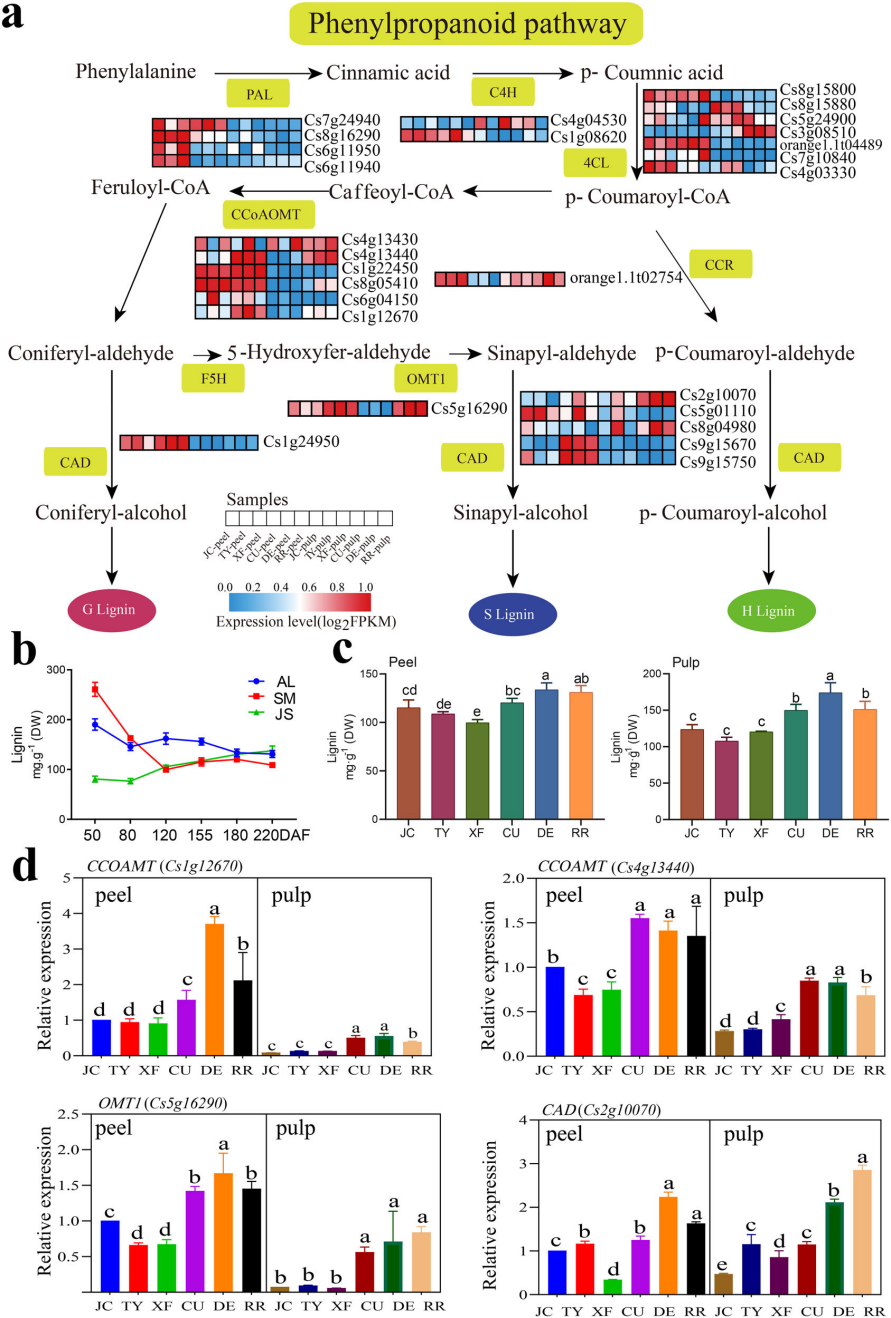
Changes in lignin content and related gene expression in CO and VO fruits. **a** Overview of phenylpropanoid pathway genes in different tissues and varieties. **b** Accumulation patterns of lignin in different sweet orange fruit tissues. AL: albedo, SM: segment membrane, JS: juice sac. **c** Lignin contents in the pulp and peel of fruits. **d** Relative expression of key genes involved in lignin synthesis in COs and VOs. Lowercase indicates statistically significant differences (*P* < 0.05)

Phenylpropanoid biosynthesis was identified as a key pathway in this study (Figs. 2, 3 and 4). As shown in Fig. 5a, most of the genes except *PALs* in this pathway were upregulated in the peel or pulp of VOs (Supplementary Table 8). The lignin content in different sweet orange (Fengjie 72-1) fruit tissues was measured, and it was found that the accumulation patterns of lignin in different tissues were not consistent. The content of lignin in the albedo (AL) and segment membrane (SM) decreased with fruit development but increased with fruit development in the juice sac (JS) (Fig. 5b). We also measured the contents of lignin in the peel and pulp of the three VO and three CO fruits used for RNA-seq. We found that the contents of lignin in the peel and pulp of VOs were significantly higher than those of COs, particularly in the pulp (Fig. 5c). Furthermore, we screened two *CCOAMTs* (*Cs1g12670, Cs4g13440*), a *CAD* gene (*Cs2g10070*), and an *OMT1* (*Cs5g16290*), all of which are involved in lignin synthesis, through qRT-PCR verification (Fig. 5d). The expression levels of these genes in VOs were significantly higher than those in COs, indicating that these genes may play crucial roles in the accumulation of lignin in VOs.

**Fig. 6:**
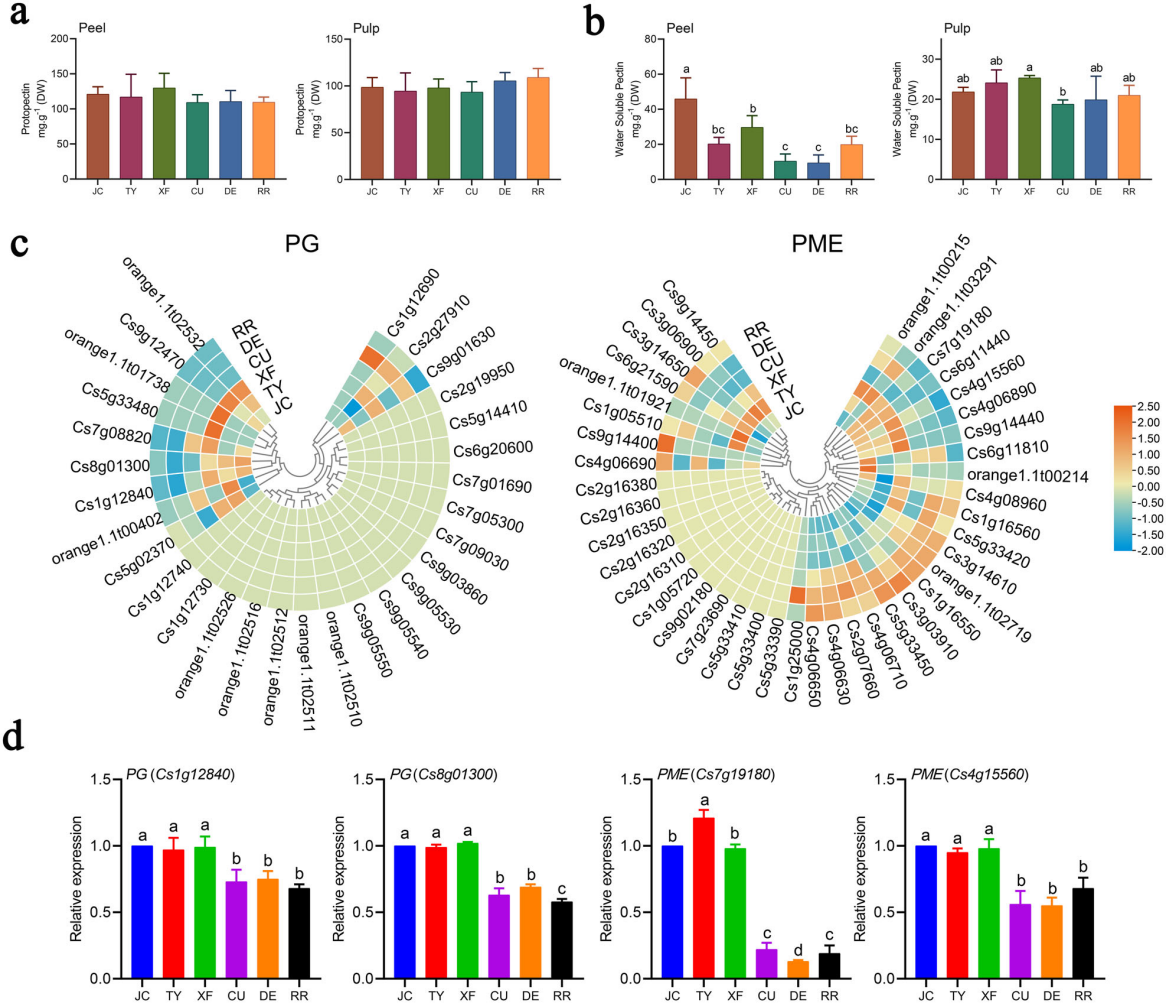
Changes in pectin content and related gene expression in CO and VO fruits. Protopectin (**a**) and water-soluble pectin (**b**) contents in the peel and pulp of fruits. **c** Heatmaps of the expression patterns of PG and PME family genes in the pulp of the six varieties. **d** Key genes involved in pectin hydrolysis in the pulp of COs and VOs. Lowercase indicates statistically significant differences (*P* < 0.05)

According to a previous study, the pectin content in fruit also affects the mastication trait of citrus fruits^29^. We measured the contents of protopectin and water-soluble pectin in three VO varieties (Cutter Valencia orange, Delta Valencia orange, and Rohde Red Valencia orange) and three CO varieties (Jincheng orange, Taoye orange, and Xianfeng orange). As shown in Fig. 6a, the protopectin contents in both the peel and the pulp were not significantly different between COs and VOs. The contents of water-soluble pectin in the COs were slightly higher than those in the VOs (Fig. 6b). Furthermore, 29 PG genes and 41 PME genes were identified from the citrus genome, and the expression patterns of these genes are shown in heatmaps, which revealed mixed expression patterns (some genes were more highly expressed in VOs, and some genes were more highly expressed in COs) (Fig. 6c; Supplementary Table 8). Among them, two PGs (*Cs8g01300* and *Cs1g12840*) and two PMEs (*Cs7g19180* and *Cs4g15560*) were more highly expressed in COs than in VOs (Fig. 6d). These results indicated that these four genes may play positive roles in the formation of citrus fruit mastication trait.

## Discussion

Valencia orange is an important late-ripening citrus variety type that is widely cultivated worldwide. However, the fruit quality of VO is worse than that of mid-ripening sweet orange, especially the pulp mastication trait. In this study, whole-genome resequencing and transcriptome analysis provided important insights into VO fruit mastication trait.

### The stress response may play important role in the formation of citrus fruit characteristics

Selective pressures accompany crop origins and diversification^30^. The CO and VO variety groups originated via natural and artificial selection processes and formed their own characteristics. In this study, according to the results of selective sweep analysis, the selected genes in both VO and CO, most of which encoded disease resistance proteins, were mainly related to the stress response (Supplementary Fig. S3; Supplementary Table S4). Moreover, according to the RNA-seq results, the DEGs and genes with different expression patterns among VOs and COs were also enriched in stress response-related biological processes, including response to stimulus, response to cold, and response to oxidative stress (Supplementary Figs. S5 and S7). These results suggested that COs and VOs have formed different stress response abilities under natural and artificial selection processes. In general, cell walls undergo lignification during plant stress^14^. For example, in soybean, salt stress enhances lignin production and restricts root growth^15^. Zeng et al. reported that the lignin content increased at low temperatures in loquat fruit^17^. In citrus, with respect to boron deficiency, Carrizo citrange is a tolerant rootstock, and trifoliate orange is a sensitive rootstock. The lignin content in the root cell walls of trifoliate orange was much higher than that in Carrizo citrange under boron deficiency stress^16^. These reports suggest that lignin or lignification may be a marker reflecting the tolerance levels of plants to various stresses. In this study, the selected genes in VOs identified by selective sweep analysis were significantly enriched in lignin biosynthetic processes (Supplementary Fig. S3b). The DEGs and genes exhibiting different expression patterns among VOs and COs during fruit ripening were also significantly enriched in lignin biosynthetic processes (Supplementary Figs. S5 and S7). In addition, the lignin content in VO fruits was significantly higher than that in CO fruits (Fig. 5c). Therefore, we suggest that the stress response may play significant role in the formation of citrus fruit characteristics during evolution, in which lignin may act as an important participant.

### Lignin may be the key component affecting the mastication trait of VO fruit

During fruit ripening, modification of the cell wall affects the horticultural characteristics of fruit, such as their hardness, brittleness, and chewiness^31^. Pectin, cellulose, and lignin are the main components of the cell wall^2,3^, and their contents may be closely related to the mastication trait of citrus fruit.

Pectin is the main component of fruit cell walls and is extensively modified in mature fruits by its involvement in fruit softening^32^. Enzymes related to pectin metabolism, such as PGs and PMEs, have received widespread attention. In *Fragaria chiloensis*, the expression and enzyme activity of PGs are significantly positively correlated with the degree of fruit softening during fruit ripening^33^. PMEs are involved in early cell wall disassembly and coordinate with PGs to participate in the degradation of pro-pectin, which is a prerequisite for PG involvement in fruit ripening^34^. Protopectin is hydrolyzed to water-soluble pectin by these cell wall modification enzymes. FJWC navel orange (*C. sinensis*) is a harder and coarser late-ripening bud sport of FJ72-1, and the content of protopectin in FJWC is higher than that in FJ72-1, while the content of water-soluble pectin is lower than that in FJ72-1^35^. In the present study, two PGs (*Cs8g01300* and *Cs1g12840*) and two PMEs (*Cs7g19180* and *Cs4g15560*) were identified as being more highly expressed in COs than in VOs (Fig. 6d). However, the protopectin contents in both the peel and the pulp were not significantly different between the COs and VOs, and the contents of water-soluble pectin showed a significant difference only among 1–2 varieties (Fig. 6a, b). These results revealed that pectin is not the critical component that results in inferior mastication trait of VO fruit, while these PG and PME genes may play partial roles in citrus fruit mastication trait formation.

Lignin content contributes to the firmness and hardness of the fruit and thus may affect fruit mastication^36^. Many studies have demonstrated that cold acclimation and freezing tolerance promote the accumulation of lignin, which further affects the quality of the fruit^5,6^, and low-temperature-grown poplar plants present increased lignin contents^37^. The fruit development and ripening time of VOs usually takes 11–12 months, so VO fruit is subjected to 3–4 months of low temperature (throughout the whole winter), while the fruit development and ripening time of COs generally takes 7–8 months; their fruit generally is subjected to only 1–2 months of low temperature. Therefore, in long-term interactions with the environment, VOs are exposed to stronger environmental stress than COs, so unique quality characteristics have developed throughout evolution. In this study, the lignin contents in the VO fruits were significantly higher than those in the COs fruits (Fig. 5c). Moreover, six genes related to phenylpropanoid/lignin biosynthesis were selected only in VOs with strong selective sweep signals, and upregulation expressed in VO fruits (Supplementary Fig. S3b, Fig. 5a; Supplementary Table S4). These results revealed that the phenylpropanoid biosynthesis pathway may play a significant role in the formation of VO fruit mastication trait. In addition, CCOAMTs, OMT1s, and CADs are important enzymes that catalyze lignin monomer synthesis. Among these enzymes, CADs function in catalyzing the last step of lignin synthesis^38,39^. In pear, inhibiting the expression of lignin biosynthesis-related genes in fruit could prevent secondary thickening of the cell wall^40^. Therefore, the expression patterns of lignin biosynthesis-related genes were analyzed, and the expression levels of these genes in VO were significantly higher than those in CO (Fig. 5d). Thus, we suggest that lignin may be the key component affecting the mastication trait of VO fruit and that lignin biosynthesis-related genes may contribute to the differences in fruit mastication trait between VO and CO fruits. Further studies are needed to elucidate the gene functions and regulatory networks involved in the formation of fruit mastication trait.

## Conclusion

An integrated analysis of the genomes and transcriptomes of VOs and COs was performed in this study. Several key biological processes and pathways were identified to play important roles in citrus fruit mastication trait formation, such as stress response-related processes and the phenylpropanoid biosynthesis pathway. Several important genes were identified in this study, including *CsERF74*, *CsNAC25*, *PGs*, *PMEs*, *CCOAMTs*, *OMT1*, and *CAD*. The results of this study provide new clues for the future investigation of the mechanisms that regulate citrus fruit mastication trait.

## Materials and methods

### Plant materials and sample collection

Whole-genome resequencing was performed on 25 sweet orange (*C. sinensis* L. Osbeck) varieties, which included 12 CO varieties and 13 VO varieties (Supplementary Table S1). After removing the varieties with similar characteristics, these 25 varieties differed in fruit quality, fruit size, fruit color, number of seeds, cultivation characteristics, maturity, and country of origin, among other characteristics (Supplementary Table S1). Young leaves were collected for genomic DNA extraction. Fruit samples of the three VO varieties (accession IDs: CO_2_, CO5, and CO11) and the three CO varieties (accession IDs: VO2, VO5, and CO7) harvested at 220 DAF were divided into peels and pulp for RNA-seq (Supplementary Table S7) and real-time quantitative PCR. Three biological replicates (two trees per replicate) were harvested per variety, and nine representative fruits were sampled from each tree. At 220 DAF, the COs were at the mature stage, while the VOs were at the coloring stage. All the trees were grown in the Citrus Resource Nursery, Chongqing Institute of Citrus Science, China.

### DNA sequencing, sequence alignment, and detection of variations

Leaves were used for genomic DNA extraction and sequenced on an Illumina HiSeq^TM^ 2500 instrument (Novogene, Beijing, China) following the Illumina protocol. The *C. sinensis* genome^41^ was used as the reference genome. The DNA resequencing data were aligned to the reference genome by BWA^42^. The alignment results were deduplicated by SAMtools^43^. The raw data have been submitted to the NCBI SRA database under accession number PRJNA687608.

SNP calling was performed using SAMtools^43^. The Bayesian model was used to detect polymorphic loci in the population, and high-quality SNPs were obtained through the following methods: the ‘mpileup’ command was used to identify SNPs with the parameters ‘-q 1 -C 50 -t SP -t DP -m 2 -F 0.002’, and after excluding SNP calling errors caused by incorrect mapping or InDels, only high-quality SNPs (coverage depth ≥3 and ≤50, RMS mapping quality ≥20, maf ≥0.05, miss≤0.1) were retained for subsequent analysis. ANNOVAR^44^ was then used to annotate the SNPs (Supplementary Table S2).

### Population genetic diversity and selective pressure analysis

The distance between populations was calculated on the basis of the obtained individual SNPs. TreeBEST-1.9.2 software was used to construct a distance matrix, and the neighbor-joining method was used to construct a phylogenetic tree^45^. Principal component analysis (PCA) was performed using GCTA software^46^. VCFtools^47^ was then used to calculate the population differentiation (*F*_*st*_), nucleotide diversity (π), and Tajima’s D values. *F*_*st*_ and θ_π_ were subsequently calculated, using a 20-kb window with a 10-kb step size.

### Linkage disequilibrium analysis

The pattern of linkage disequilibrium (LD) was compared using high-quality SNPs and calculated with Haploview^48^. The coefficient (*r*^2^) between pairwise SNPs was calculated and the parameters were set as: ‘-n -dprime-minMAF 0.05’. The average *r*^2^ value was calculated for pairwise markers within a 5000-kb window and averaged across the whole genome.

### Selective sweep analysis

Selective sweep analysis was performed on the basis of the F_*st*_ and θ_π_ values. Both the threshold values of F_*st*_ and the θπ ratio used to identify selected regions were in the top 5%. The process was performed as described by Xia et al.^49^. Genome-wide distribution of fixation index (F_*st*_) values and θ_π_ ratios were used for the defined group pairs. The F_*st*_ values were Z -transformed as follows: Z(F_*st*_) = (F_*st*_−µF_*st*_)/σF_*st*_, in which µF_*st*_ is the mean F_*st*_ and σF_*st*_ is the standard deviation of F_*st*_. The θπ ratios were then log_2_ transformed. Subsequently, we estimated and ranked the empirical percentiles of the Z(F_*st*_) and log_2_(θ_π_ ratio) values in each window. We considered windows with the top 5% of Z(F_*st*_) and log_2_(θ_π_ ratio) values simultaneously as candidate outliers under strong selective sweeps.

### RNA-seq, data processing, and gene functional annotation

Twelve samples of two fruit tissues (peel and pulp) from six varieties were used for RNA-seq. Each sample was sequenced, which included three biological replicates. Total RNA isolation was performed, as described previously^50^. The data for a total of 36 transcriptome profiles were obtained by using the Illumina HiSeq^TM^ 4000 sequencing platform at Personalbio (Shanghai, China) (Supplementary Table S7). The raw RNA-seq data have been uploaded to the Gene Expression Omnibus (GEO) database of the NCBI (accession number: GSE164142). The *C. sinensis* genome^41^ was used as the reference genome. Data processing and gene functional annotation were performed as described in our previous study^51,52^.

### Real-time quantitative PCR

Relative gene expression was detected by real-time quantitative PCR, which was performed according to the methods of our previous study^53^. The endogenous reference gene used was *CsActin*^54^. The primers used for each gene in this study are listed in Supplementary Table S9.

### Determination of the contents of lignin and pectin

Approximately 5.0 g of peel and pulp tissue from each variety was dried to a constant weight at 80 °C, ground into powder using a mortar, and passed through a 40-mesh sieve. The total lignin content of 20 mg of peel and pulp tissue was determined in accordance with the protocol of the Plant Lignin Content Kit (COMIN, MZS-1-G), which was purchased from Suzhou Comin Biotechnology Co., Ltd. (China). Approximately 3.0 g of peel and pulp tissue from each variety were used for pectin determination, as described by Lei et al.^29^.

## Supplementary information

Supplementary information

Figure S1. Statistical distribution of SNP mutation type (a), SNP support read number (b), SNP quality (c), and neighboring SNP distance (d)

Figure S2. Tajima'D test of VO and CO based on intraspecies polymorphism (a) and decay of linkage disequilibrium of VO and CO (b)

Figure S3. The GO term (biological process) enrichment analysis of selected genes from CO (a) and VO (b)

Figure S4. Global analysis of the 36 fruit transcriptomes

Figure S5. The GO term (biological process) enrichment analysis of DEGs among COs and VOs identified from peel tissue (a) and pulp tissue (b)

Figure S6. Coexpression modules identified by WGCNA

Figure S7. The GO term (biological process) enrichment analysis of the genes from blue (a), red (b), and brown (c) modules

Table S1. Overview of sample information and resequencing statistics

Table S2. Distribution of SNPs within various Citrus sinensis genomic regions

Table S3. Screened group-specific SNP molecular markers

Table S4. Annotation results of part of genes screened in CO and VO

Table S5. The results of GO term enrichment analysis

Table S6. The results of KEGG term enrichment analysis

Table S7. Overview of sample information and RNA-seq statistics

Table S8. A list of key genes identified by RNA-seq

Table S9. The primers used in this study

## Data Availability

The genome resequencing data that support the findings of this study have been deposited in the NCBI SRA database under accession number PRJNA687608. The raw RNA-seq data that support the findings of this study have been deposited in the Gene Expression Omnibus (GEO) of the NCBI under accession number GSE164142.
