# Supplementary material for: Genomic and transcriptomic analyses of Citrus sinensis varieties provide insights into Valencia orange fruit mastication trait formation
Source: Hortic Res. 2021 Oct 1;8:218. doi: 10.1038/s41438-021-00653-5 (PMC8484299; doi:10.1038/s41438-021-00653-5)
Supplement: Supplementary file 1 — Supplementary information [file 41438_2021_653_MOESM1_ESM.docx]

**Supplementary information**

**Figure S1. Statistical distribution of SNP mutation type (a), SNP support read number (b), SNP quality (c) and neighbouring SNP distance (d).**

**Figure S2. Tajima'D test of VO and CO based on intraspecies polymorphism (a) and decay of linkage disequilibrium of VO and CO (b).**

**Figure S3. The GO term (biological process) enrichment analysis of selected genes from CO (a) and VO (b).**

The enrichment results of selected genes are shown in the “TreeMap” view. Clusters are shown with different colors and rectangles. The *p*-value is used to adjust the size of the rectangles.

**Figure S4. Global analysis of the 36 fruit transcriptomes.**

(a) Cluster dendrogram showing global relationships between biological replicates and among different varieties and tissues. The *y*-axis is the degree of variance. (b) Principal component analysis (PCA). (c) Verification of DEGs with qRT-PCR. Below the bar graph is the gene expression pattern obtained by transcriptome sequencing. Each sample contains 4 replicates. In all figures to follow, TY means Taoye Orange, JC means Jincheng orange, XF means Xianfeng orange, CU means Cutter Valencia Orange, DE means Delta Valencia Orange, and RR means Rohde Red Valencia Orange.

**Figure S5. The GO term (biological process) enrichment analysis of DEGs among COs and VOs identified from peel tissue (a) and pulp tissue (b).**

The enrichment results of selected genes are shown in the “TreeMap” view. Clusters are shown with different colors and rectangles. The *p*-value is used to adjust the size of the rectangles. DEG: differential expressed gene.

**Figure S6. Coexpression modules identified by WGCNA.**

(a) Hierarchical cluster tree showing coexpression modules. (b) Eigengene expression profiles of the coexpression modules.

**Figure S7. The GO term (biological process) enrichment analysis of the genes from blue (a), red (b) and brown (c) modules.**

The enrichment results of selected genes are shown in the “TreeMap” view. Clusters are shown with different colors and rectangles. The *p*-value is used to adjust the size of the rectangles.

**Table S1.** Overview of sample information and resequencing statistics.

**Table S2.** Distribution of SNPs within various Citrus sinensis genomic regions.

**Table S3.** Screened group-specific SNP molecular markers.

**Table S4.** Annotation results of part of genes screened in CO and VO.

**Table S5.** The results of GO term enrichment analysis.

**Table S6.** The results of KEGG term enrichment analysis.

**Table S7.** Overview of sample information and RNA-seq statistics.

**Table S8.** A list of key genes identified by RNA-seq.

**Table S9.** The primers used in this study.


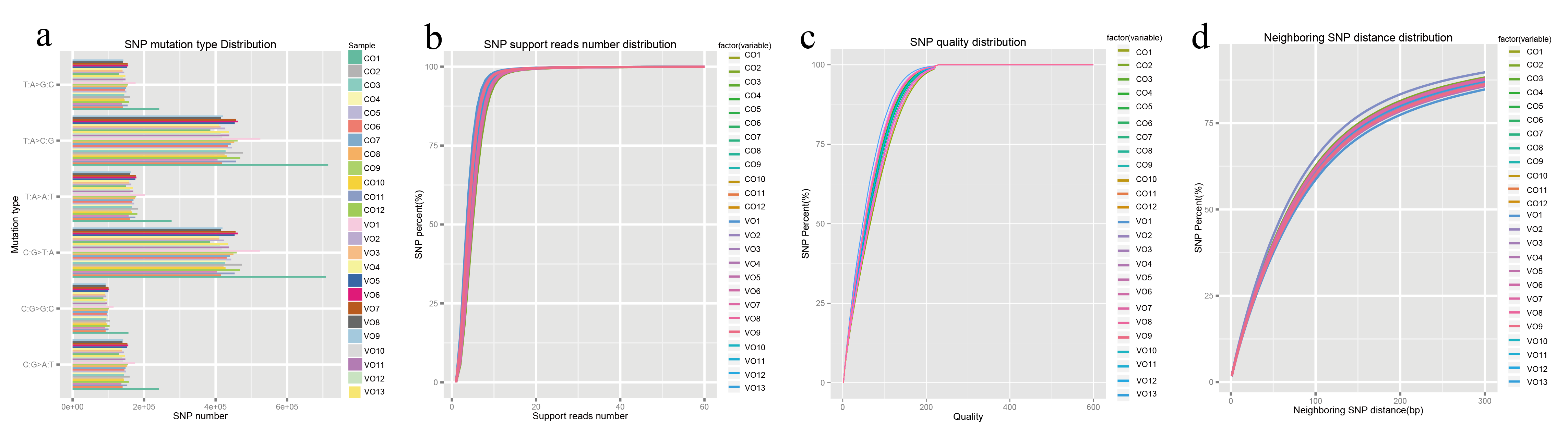


**Figure S1.** Statistical distribution of SNP mutation type (a), SNP support read number (b), SNP quality (c) and neighbouring SNP distance (d).


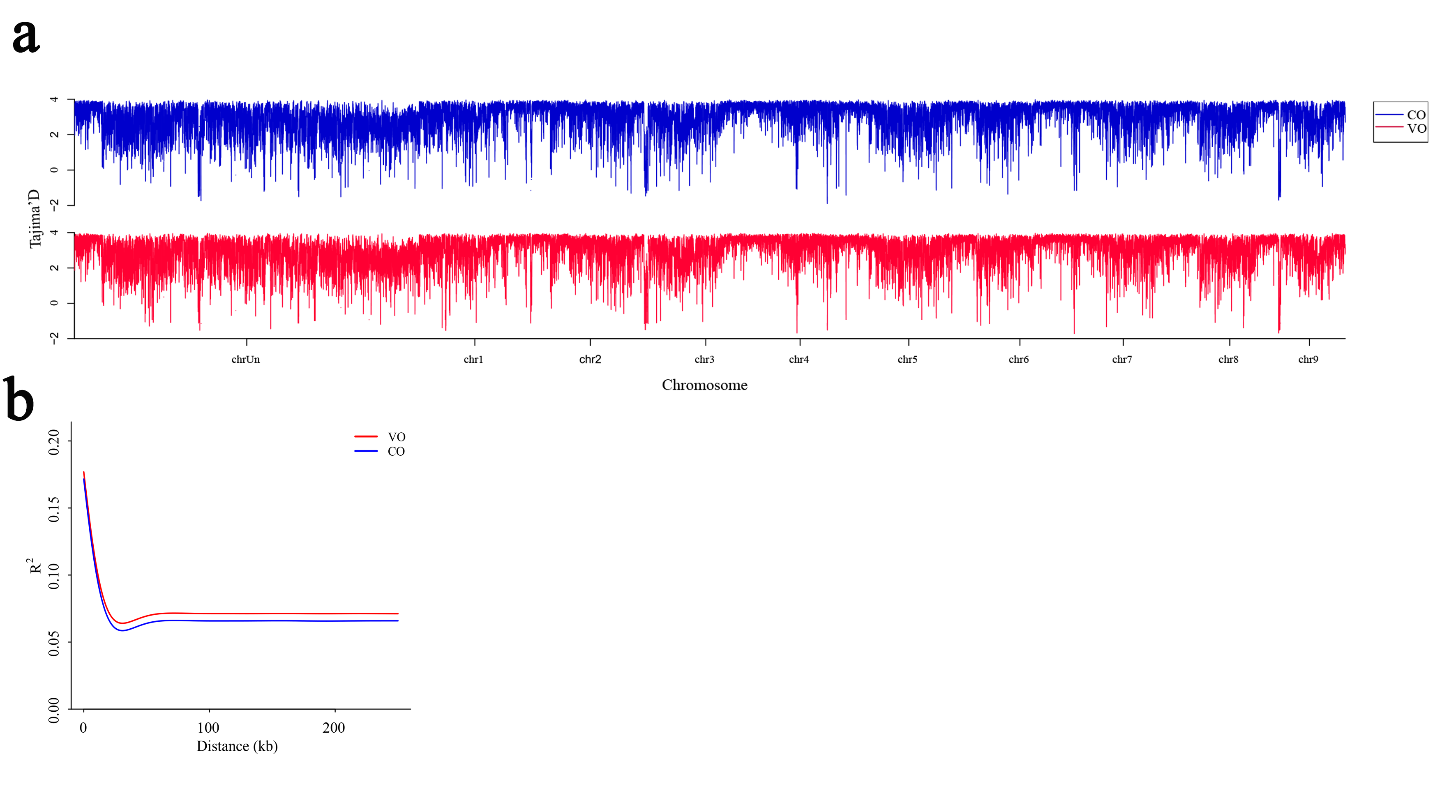


**Figure S2. Tajima'D test of VO and CO based on intraspecies polymorphism (a) and decay of linkage disequilibrium of VO and CO (b).**


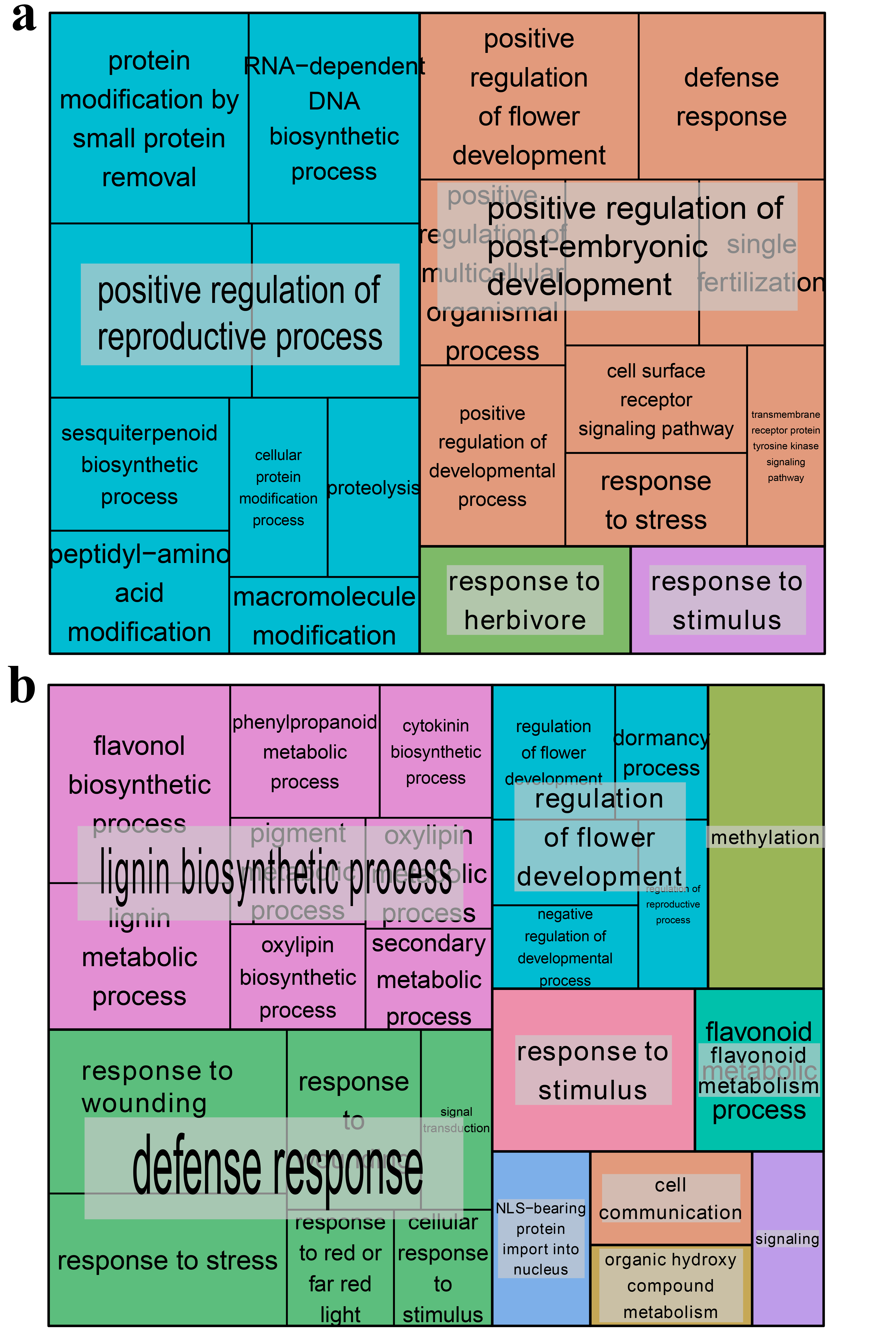


**Figure S3. The GO term (biological process) enrichment analysis of selected genes from CO (a) and VO (b).**

The enrichment results of selected genes are shown in the “TreeMap” view. Clusters are shown with different colors and rectangles. The *p*-value is used to adjust the size of the rectangles.


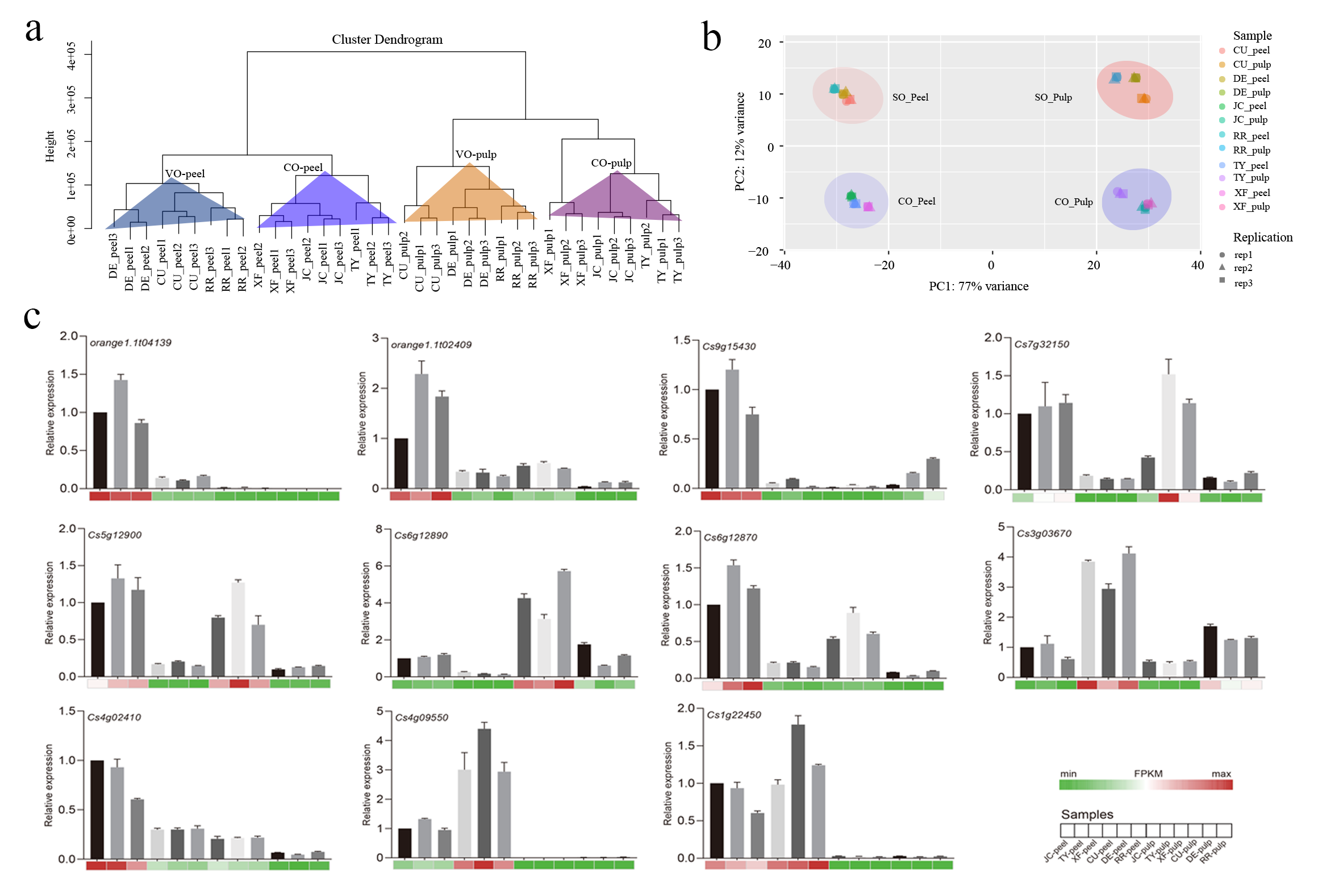


**Figure S4. Global analysis of the 36 fruit transcriptomes.**

(a) Cluster dendrogram showing global relationships between biological replicates and among different varieties and tissues. The *y*-axis is the degree of variance. (b) Principal component analysis (PCA). (c) Verification of DEGs with qRT-PCR. Below the bar graph is the gene expression pattern obtained by transcriptome sequencing. Each sample contains 4 replicates. In all figures to follow, TY means Taoye Orange, JC means Jincheng orange, XF means Xianfeng orange, CU means Cutter Valencia Orange, DE means Delta Valencia Orange, and RR means Rohde Red Valencia Orange.


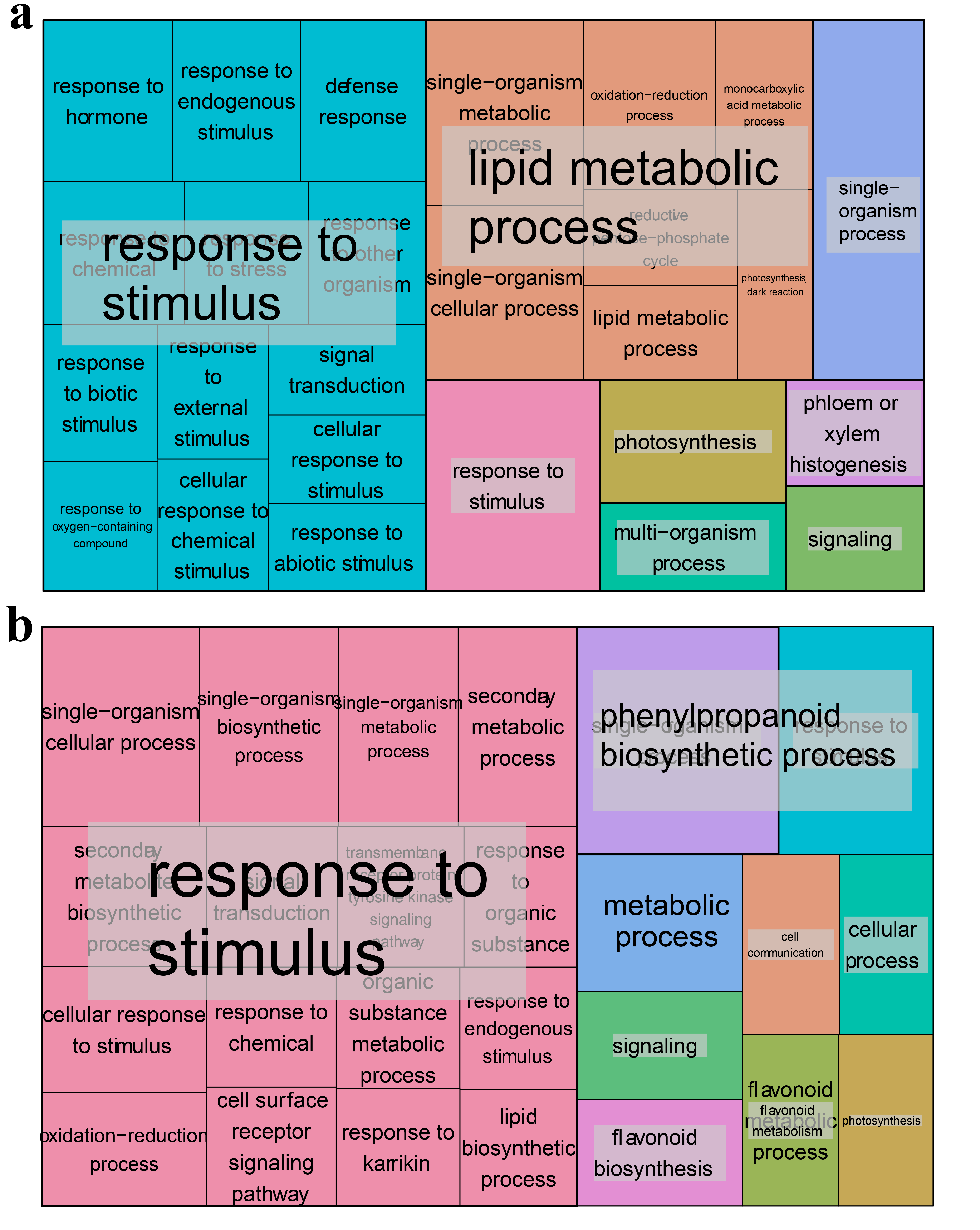


**Figure S5. The GO term (biological process) enrichment analysis of DEGs among COs and VOs identified from peel tissue (a) and pulp tissue (b).**

The enrichment results of selected genes are shown in the “TreeMap” view. Clusters are shown with different colors and rectangles. The *p*-value is used to adjust the size of the rectangles. DEG: differential expressed gene.


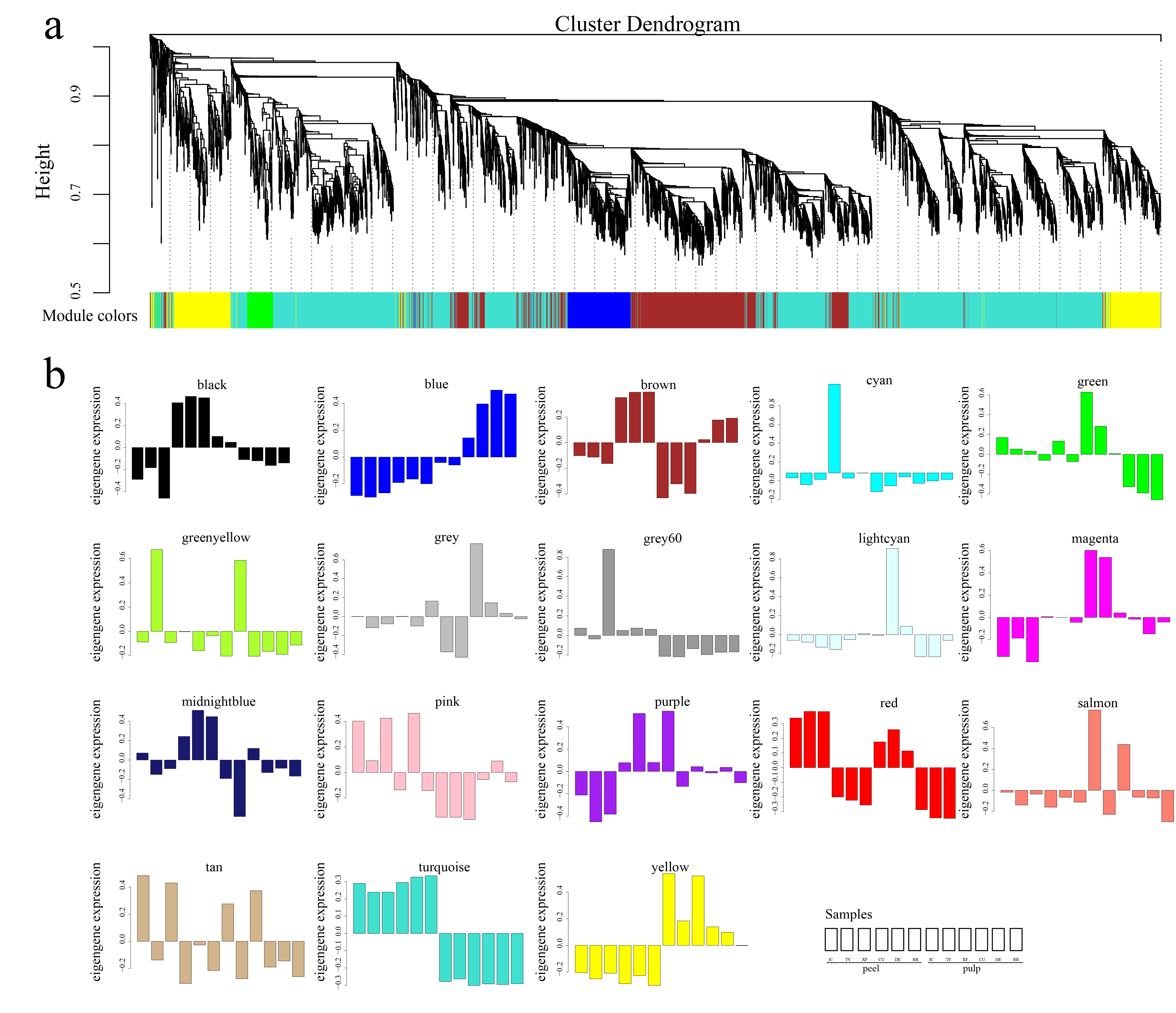


**Figure S6. Coexpression modules identified by WGCNA.**

(a) Hierarchical cluster tree showing coexpression modules. (b) Eigengene expression profiles of the coexpression modules.

**
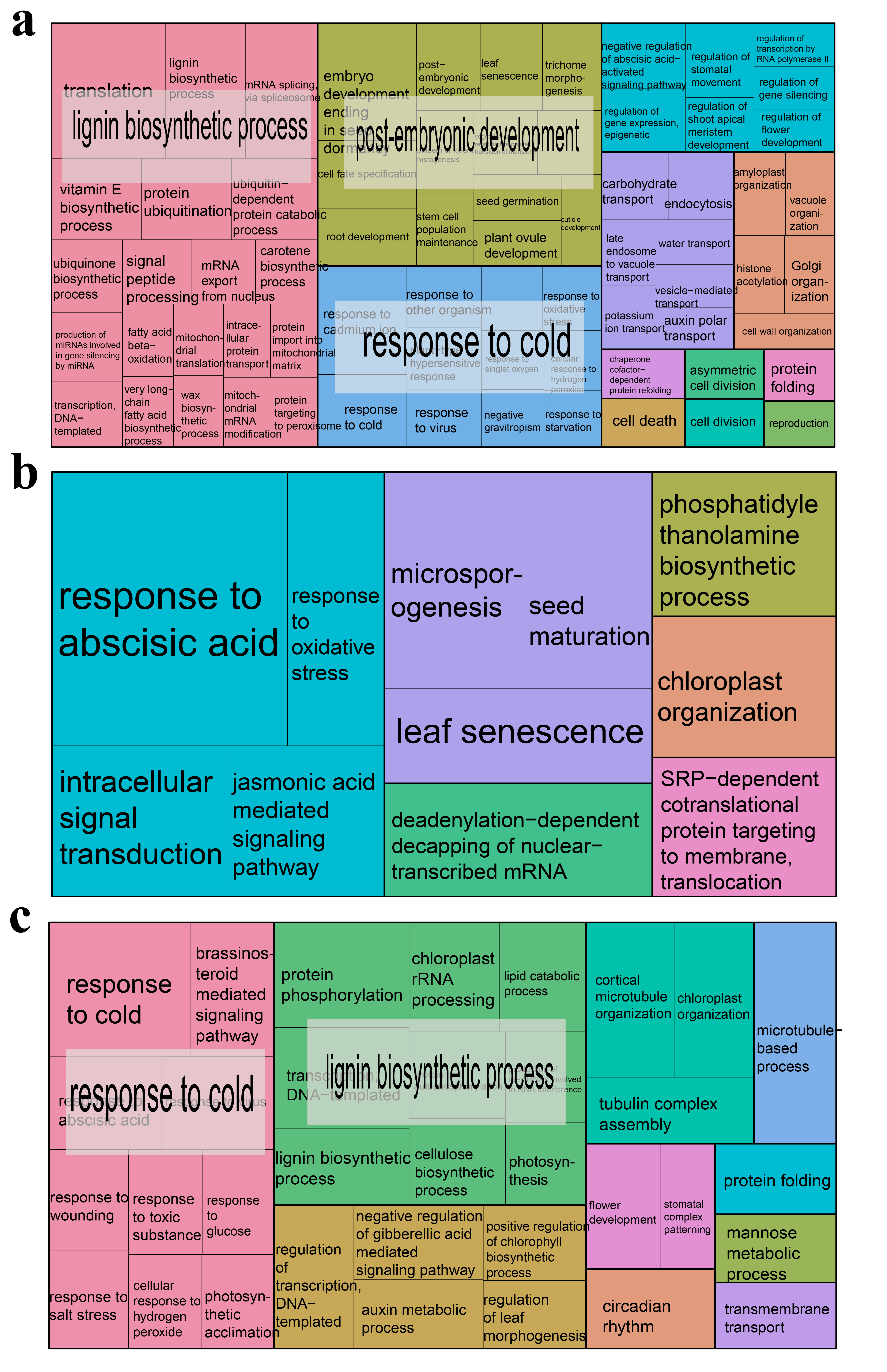
**

**Figure S7. The GO term (biological process) enrichment analysis of the genes from blue (a), red (b) and brown (c) modules.**

The enrichment results of selected genes are shown in the “TreeMap” view. Clusters are shown with different colors and rectangles. The *p*-value is used to adjust the size of the rectangles.
