# Supplementary figures and images for: Genomic and transcriptomic analyses of Citrus sinensis varieties provide insights into Valencia orange fruit mastication trait formation
Source: Hortic Res. 2021 Oct 1;8:218. doi: 10.1038/s41438-021-00653-5 (PMC8484299; doi:10.1038/s41438-021-00653-5)

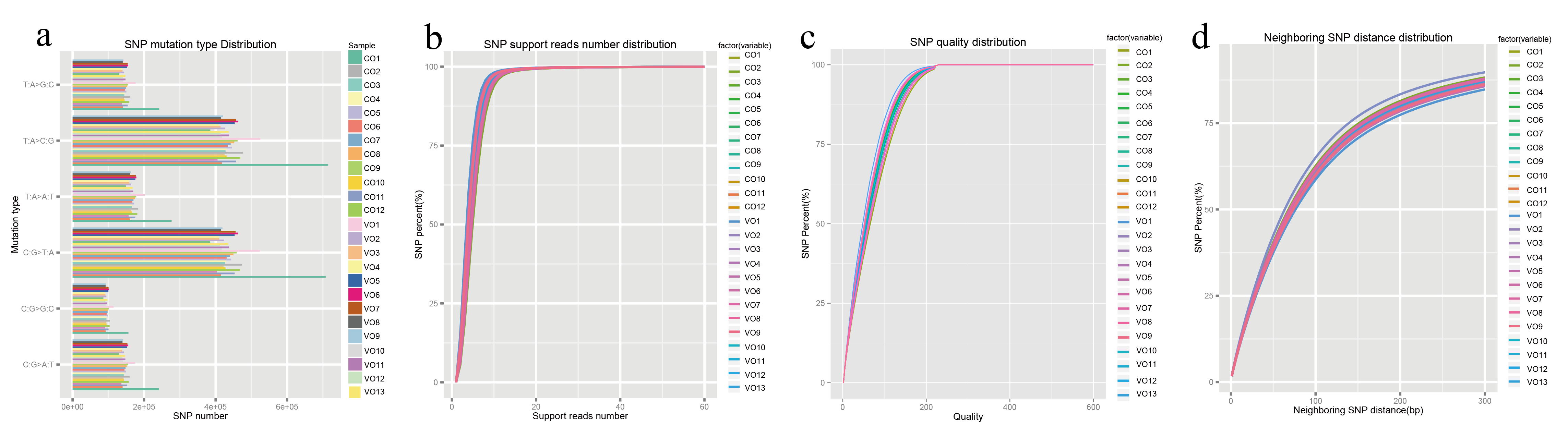

Supplement: Supplementary file 2 — Figure S1. Statistical distribution of SNP mutation type (a), SNP support read number (b), SNP quality (c), and neighboring SNP distance (d) [file 41438_2021_653_MOESM2_ESM.tif]

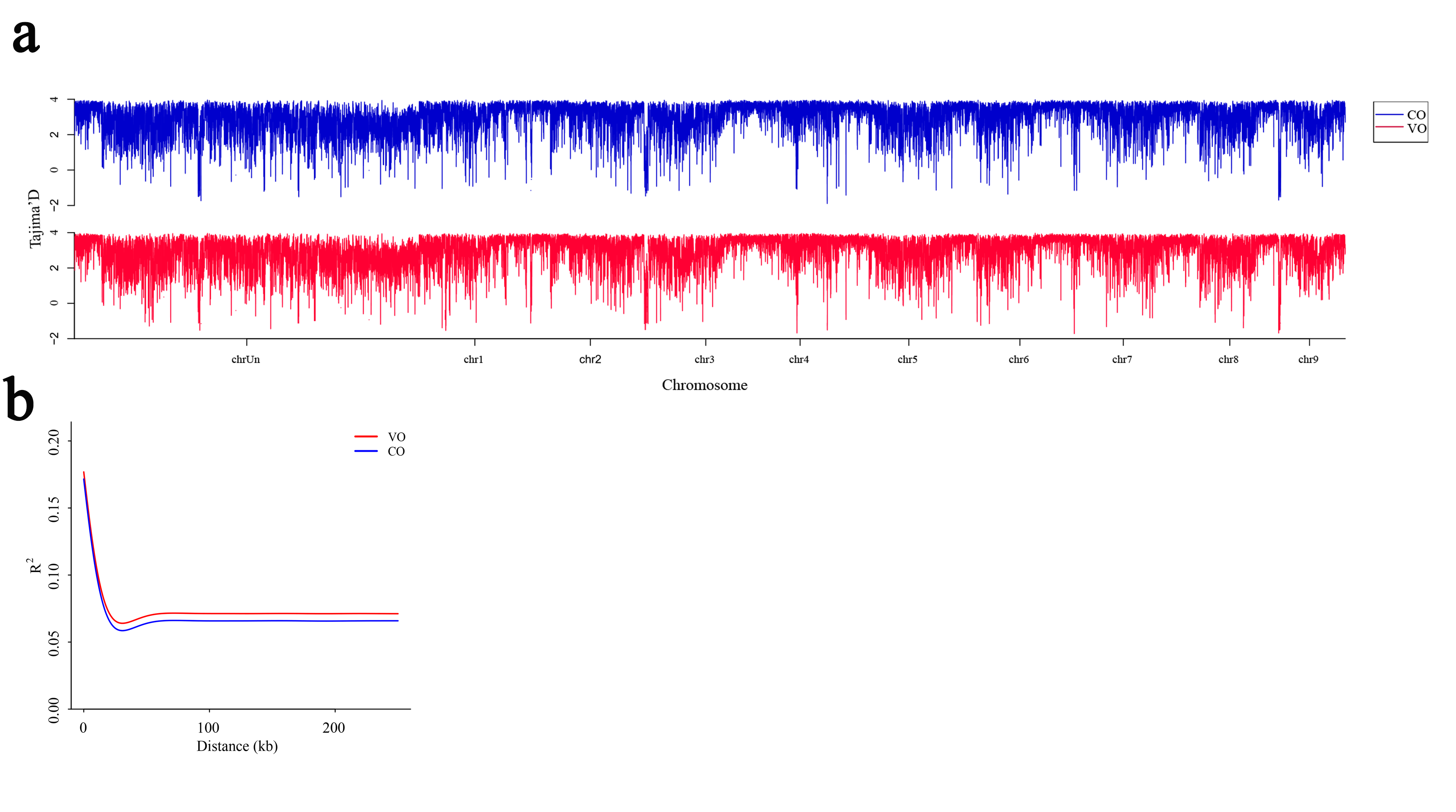

Supplement: Supplementary file 3 — Figure S2. Tajima'D test of VO and CO based on intraspecies polymorphism (a) and decay of linkage disequilibrium of VO and CO (b) [file 41438_2021_653_MOESM3_ESM.tif]

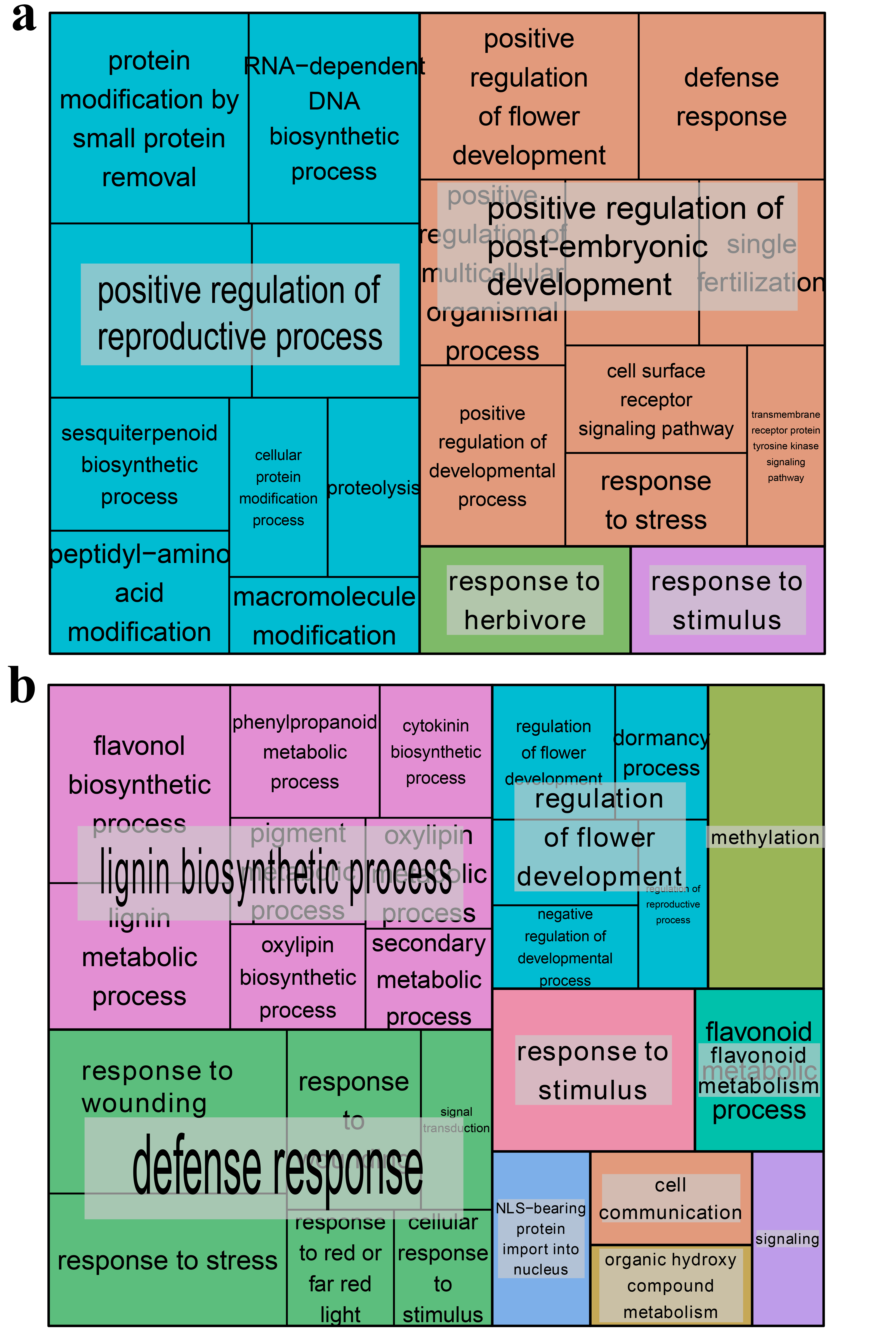

Supplement: Supplementary file 4 — Figure S3. The GO term (biological process) enrichment analysis of selected genes from CO (a) and VO (b) [file 41438_2021_653_MOESM4_ESM.tif]

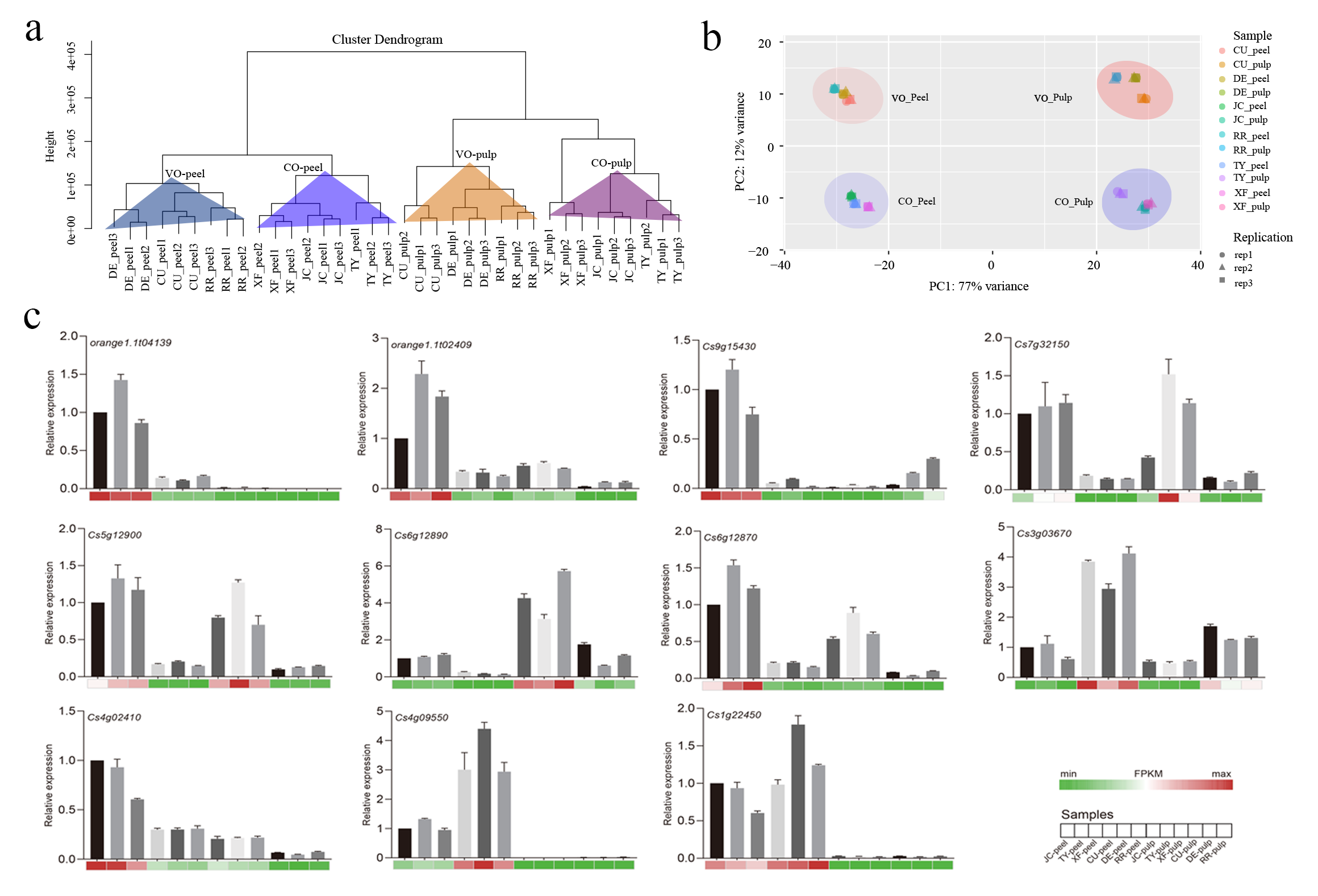

Supplement: Supplementary file 5 — Figure S4. Global analysis of the 36 fruit transcriptomes [file 41438_2021_653_MOESM5_ESM.tif]

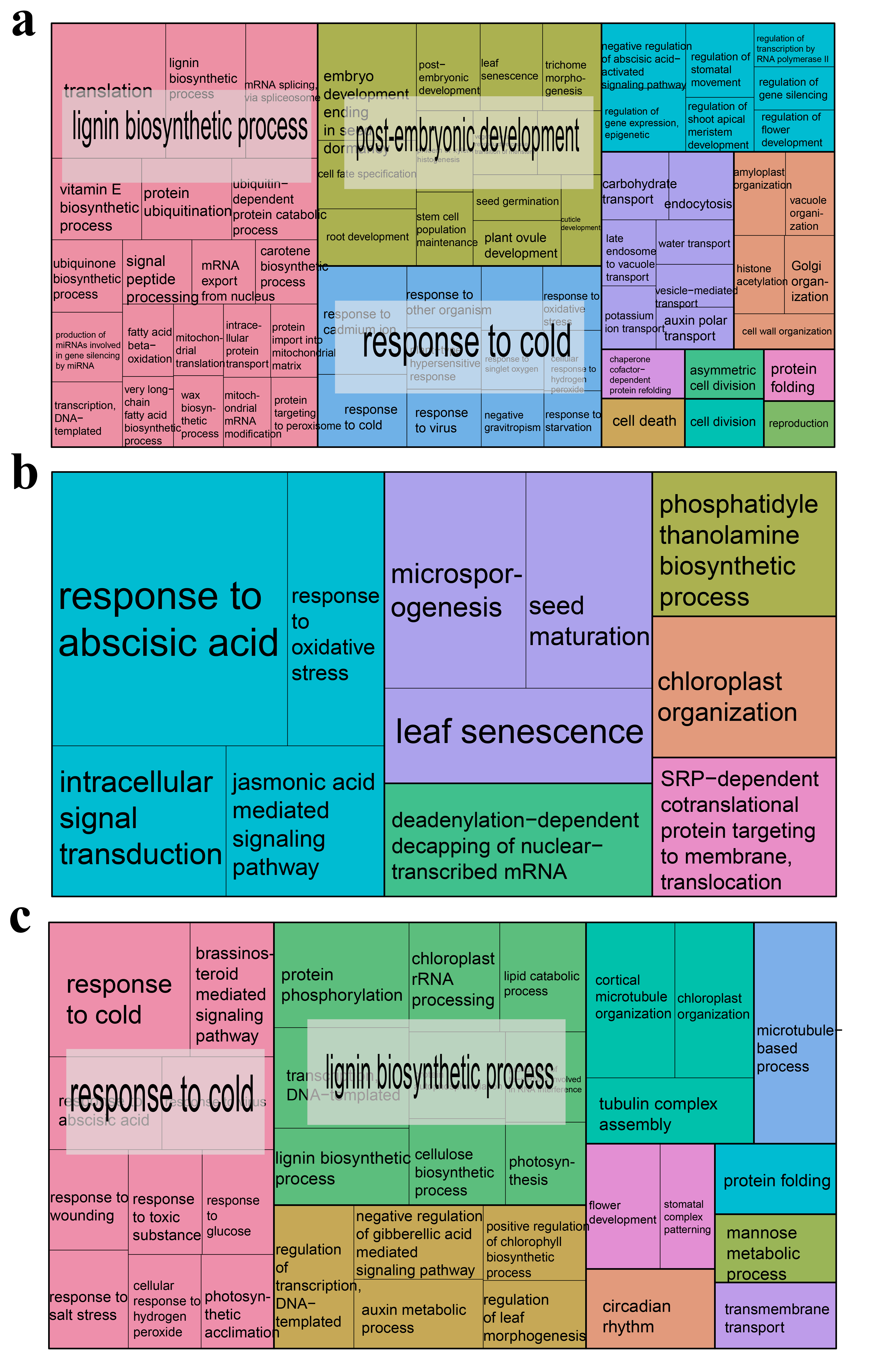

Supplement: Supplementary file 6 — Figure S5. The GO term (biological process) enrichment analysis of DEGs among COs and VOs identified from peel tissue (a) and pulp tissue (b) [file 41438_2021_653_MOESM6_ESM.tif]

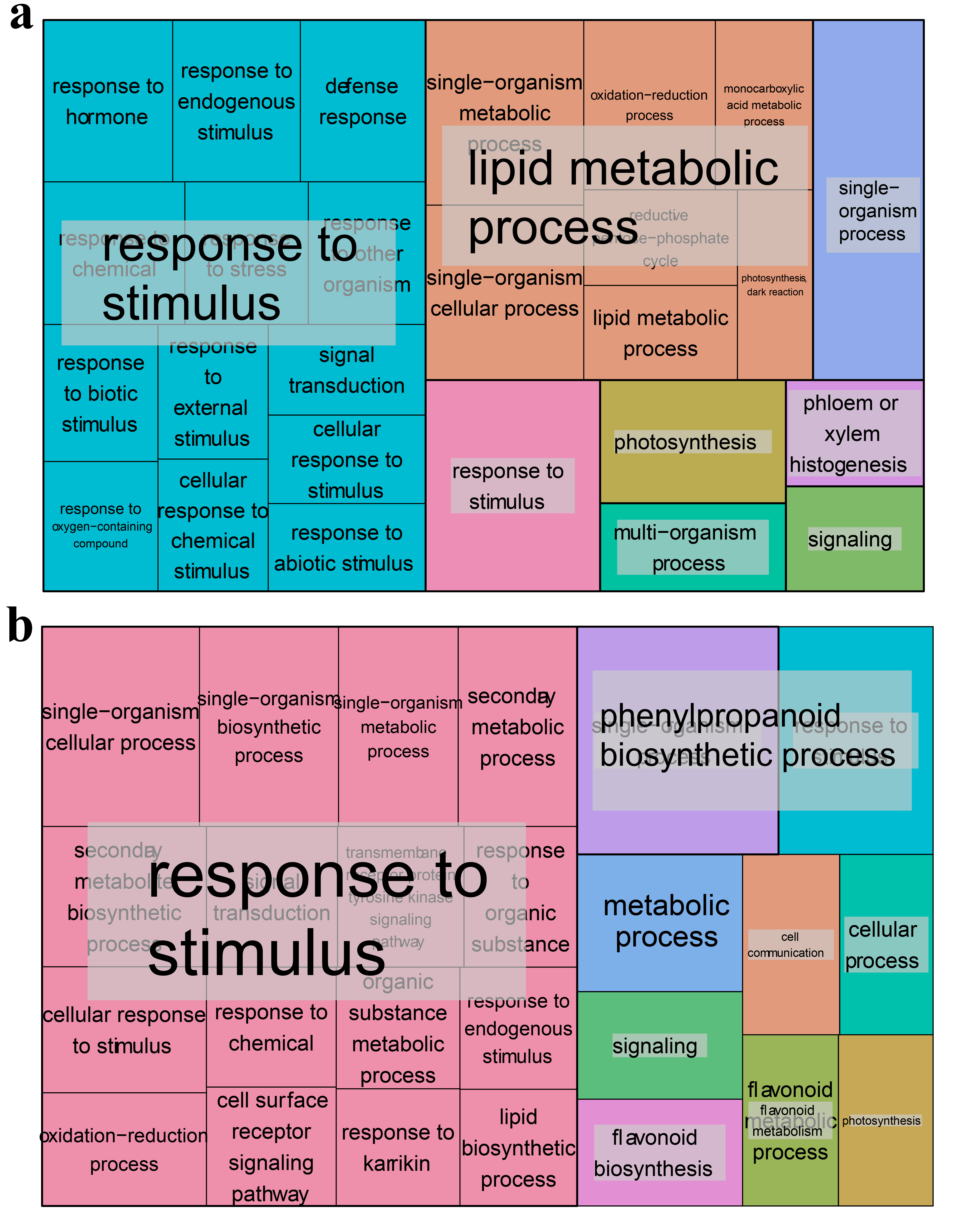

Supplement: Supplementary file 7 — Figure S6. Coexpression modules identified by WGCNA [file 41438_2021_653_MOESM7_ESM.tif]

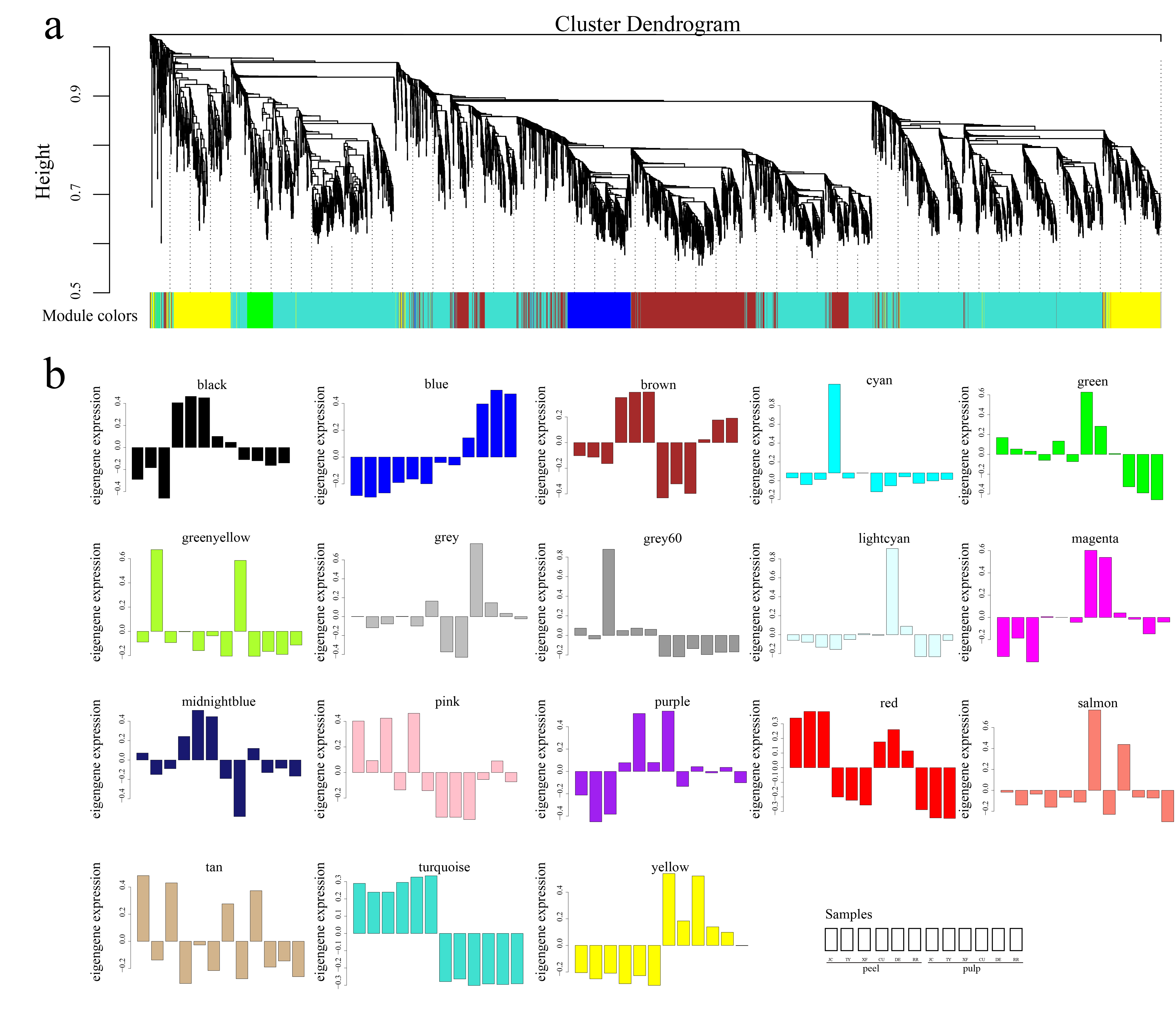

Supplement: Supplementary file 8 — Figure S7. The GO term (biological process) enrichment analysis of the genes from blue (a), red (b), and brown (c) modules [file 41438_2021_653_MOESM8_ESM.tif]
